# Supplementary material for: Health-related behavioral changes during the COVID-19 pandemic. A comparison between cohorts of French and Italian university students
Source: PLOS Glob Public Health. 2023 Sep 8;3(9):e0002298. doi: 10.1371/journal.pgph.0002298 (PMC10490880; doi:10.1371/journal.pgph.0002298)
Supplement: S1 Table — Percentages (Modal values in bold) or absolute values. (DOCX) [file pgph.0002298.s001.docx]

**S1 Table**

**S1 Table. Dietary habits of the respondents compared by participant cohort. Percentages (Modal values in bold) or absolute values.**

| **Item** | **France**  (n=400) | **Italy**  (n=167) |  | **Item** | **France**  (n=400) | **Italy**  (n=167) |
| --- | --- | --- | --- | --- | --- | --- |
| **B1- Habit of 3 meals a day**  Yes  No  **B2 - Average time dedicated to breakfast**  Less than 5 minutes  Between 5 and 10 minutes  More than 10 minutes  **B3 - Practice of having breakfast in front of the** **TV**  Yes  No  **B4 - Frequency of meals away from home (restaurant, fast food, kiosks and bars)**  Never or rarely  1 to 4 times a week  More than 4 times a week  **B5 - Average time dedicated to lunch**  Less than 10 minutes  Between 10 and 15 minutes  More than 15 minutes  **B6 - Practice of having lunch in front of the TV**  Yes  No  **B7 - Sweetened drinks consumed daily**  None  1 can of 33 cl  More than a 33 cl can  **B8 - Types of fruit consumed daily**  None  1 type of fruit  Different kinds of fruit  **B9 - Average time dedicated to dinner**  Less than 10 minutes  Between 10 and 15 minutes  More than 15 minutes | **66,6**  33,4  35,4  **41,0**  23,6  24,2  **75,8**  37.2  **61.3**  1.5  7,3  40,2  **52,5**  29,5  **70,5**  **56,9**  31,0  12,1  22,7  **49,7**  27,5  4,3  32,1  **63,6** | **93,4**  6,6  20,4  **46,7**  32,9  25,1  **74,9**  **65.9**  34.1  0.0  2,4  25,7  **71,9**  **62,9**  37,1  **89,8**  9,0  1,2  14,4  **50,9**  34,7  2,4  22,2  **75,4** |  | **B10 - Practice of having dinner in front of the TV**  Yes  No  **B11 - Regularity of consumption of dairy products (yogurt, etc.)**  Yes  No  **B12 - Practice of eating while watching TV**  Yes  No  **B13 - Type of food consumed while watching TV**  Sweets  Potato chips  Fruit  Dairy products  **B14 - Practice of snacking between meals**  Yes  No  **B15 - Regularity of vegetable consumption**  Yes  No  **B16 - Types of vegetables consumed daily**  1 type of vegetable only  2 types of vegetables  More than 2 types of vegetables  **B17 - Being on a diet**  Yes  No  **B18 - Possible dieting frequency**  1 time  2 or 3 times  More than 3 times | **50,3**  49,7  **82,0**  18,0  40,8  **59,2**  (n=290)  **52,6**  24,6  8,6  14,2  **61,8**  38,2  **71,4**  28,6  (n=302)  **38,7**  32,1  29,1  25,6  **74,4**  (n=108)  **39,8**  38,0  22,2 | **66,5**  33,5  **86,2**  13,8  **55,1**  44,9  (n=148)  19,2  7,7  **42,3**  30,8  **87,4**  12,6  **88,6**  11,4  (n=148)  34,2  **47,7**  17,4  46,1  **53,9**  (n=77)  36,4  **42,8**  20,8 |
