# Supplementary material for: Health-related behavioral changes during the COVID-19 pandemic. A comparison between cohorts of French and Italian university students
Source: PLOS Glob Public Health. 2023 Sep 8;3(9):e0002298. doi: 10.1371/journal.pgph.0002298 (PMC10490880; doi:10.1371/journal.pgph.0002298)
Supplement: S2 Table — Percentages (Modal values in bold) or absolute values. (DOCX) [file pgph.0002298.s002.docx]

**S2 Table**

**S2 Table. Physical activity practices of the respondents compared by participant cohort (IFIS). Percentages (Modal values in bold) or absolute values.**

| **Item** | **France** (n=400) | **Italy** (n=167) |
| --- | --- | --- |
| **C1- Your general physical fitness is**  Very poor  Poor  Average  Good  Very good  **C2 - Your capacity to do exercise for a long time**  Very poor  Poor  Average  Good  Very good  **C3 - Your muscle strength is**  Very poor  Poor  Average  Good  Very good  **C4 - Your speed is**  Very poor  Poor  Average  Good  Very good  **C5 - Your agility is**  Very poor  Poor  Average  Good  Very good  **C6 - Your flexibility is**  Very poor  Poor  Average  Good  Very good  **IFIS physical state scale (summary of items C1-C6 on a scale of 0-1)**  Average value  Standard deviation  **C7 – Assessment of sports practice level (scale 0-5) of parents, friends or acquaintances**  Average value  Standard deviation  **C8 - Doing sports as a professional**  Yes  No  **C9 - Membership in a sports club (registered)**  Yes  No | 4.8  11.7  36.1  **36.2**  11.2  11.0  14.1  23.3  **32.2**  19.4  3.1  11.8  **41.4**  36.0  7.7  5.6  14.6  **38.2**  32.9  8.7  1.3  8.5  31.5  **46.9**  11.8  8.7  22.4  28.6  **29.1**  11.2  0.59  0.19  2.55  1.70  3.8  **96.2**  40.8  **59.2** | 0.6  10.2  **35.3**  34.1  19.8  2.4  7.2  18.6  33.5  **38.3**  1.8  10.8  27.5  **41.3**  18.6  3.0  11.4  35.9  **37.1**  12.6  1.8  7.8  29.9  **38.9**  21.6  5.4  17.4  **33.5**  26.9  16.8  0.66  0.19  3.10  1.35  6.0  **94.0**  38.9  **61.1** |
