# Supplementary material for: Health-related behavioral changes during the COVID-19 pandemic. A comparison between cohorts of French and Italian university students
Source: PLOS Glob Public Health. 2023 Sep 8;3(9):e0002298. doi: 10.1371/journal.pgph.0002298 (PMC10490880; doi:10.1371/journal.pgph.0002298)
Supplement: Attachment — Submitted filename: Response to Reviewers.docx [file pgph.0002298.s003.docx]

**Response to Reviewers**

5. Review Comments to the Author

Reviewer #1: The current study aimed to investigate the impact of the COVID-19 pandemic on choices and lifestyle changes in a sample of Italian and French university students.
The paper provides a modest scientific contribution to the wide panorama of studies investigating the general population's health and quality of life during the Covid-19 pandemic.
The topic is interesting enough, but significant changes and explanations should be made to improve the quality of the manuscript, especially in conceptual terms, for his acceptance.

Abstract section
In the ‘abstract section’, the Authors should briefly describe the survey period, the procedures, and the methodology used to recruit, assess, and analyze the considered variables. The results are summarized without any statistical data, and there are not conclusions concerning future clinical-social implications. The Authors should add them.

In the ‘abstract section’ the authors has included the survey period, the procedures and the metodology used to recruit, assess and analyze the considered variables.

The results have been integrated with statistical data. Conclusions with clinical-social implications have been added.

The keyword "mental health" could be entered

The keyword "mental health" has been entered.

Introduction section
The bibliography in the ‘introduction section is appropriate and updated but very poor. However, it would be useful to mention, for example, recent international studies (1, 2, 3, 4, 5, listed below) on the impact of Covid -19 pandemic on college students.
Some authors focused on psychological, emotional, and cognitive correlates of social confinement in a sample of college students, integrating qualitative and quantitative analyses to identify potential predictors of traumatic distress during Covid -19 and low academic performance during Covid -19. The Authors identified the thinking style “all or nothing” as the strongest predictor of traumatic distress in a sample of university students. Another recent UK population survey showed young adults coping poorly, and pre-existing mental health conditions were significantly associated with suicidal thoughts and self-harm.
An Australian study showed that loneliness moderated the mediating effect of a resilient mindset in the coronavirus stress and depressive symptoms association among college students.

The authors do not explain their study's objectives and/or hypotheses at the end of the introduction.

In the Introduction section, the bibliography has been implemented with recent international studies on the impact of Covid-19 pandemic on college students.

At the end of the Introduction section the study’s objectives and hypotheses have been explained.

Materials and methods section
The methods and procedures are well described. The tables are exhaustive for data presentation. The Authors should clarify how focus groups were organized to develop the survey (questions/items). It is not clear who conducted this investigation. Is this perhaps, for example, a sort of Listening and Counselling Service for college students? The Authors should make this clear.

In the material and methods section the focus group organization for the development of the reseach has been clarified. The conduction of the investigation has been clarified.

Discussion and Conclusions section
The discussion is incomplete: there are no comments on the results with comparisons with other surveys conducted during the pandemic, strengths and limitations of the study, study conclusions, and clinical/social future implications. Authors should make the suggested enrichments.

The discussio has been completed. Comparitions with other surveys, strenght and limitations of the study have been added.

The authors make no mention of the University Listening and Consultation Services, a well-represented reality in the academic world, to help students cope with the emotional distress related to academic difficulties.

The authos mentioned the University Listening and Consulting Services and their functions.

Within the future clinical implications, it would be useful to emphasize the need for the implementation of psychological interventions, even digital, to improve the mental health of vulnerable young subgroups during a global health emergency as the Covid-19 pandemic, and contain, as far as possible, the evolution of psychopathological profiles represents a fundamental challenge (6, 7 listed below)

Future clinical implications as the need for the implementation of relational and psychological interventions, even digital, to improve mental health of subgroups during the global health emergency have been added.

Overall, the manuscript's clarity is good enough in the language style, syntax, and sentence construction.

In the ‘Conclusions section,’ the Authors should report how their study contributes to achieving the Global Goals of the WHO 2030 Agenda for Sustainable Development.

The article contributions to the achievement of the Global Goals of the 2030 Agenda for Sustainable Development have been added.

1. <https://pubmed.ncbi.nlm.nih.gov/33384623/>
2. <https://pubmed.ncbi.nlm.nih.gov/36377653/>
3. <https://pubmed.ncbi.nlm.nih.gov/36339876/>
4. <https://www.ncbi.nlm.nih.gov/pmc/articles/PMC8718341/>
5. <https://pubmed.ncbi.nlm.nih.gov/34526153/>
6. <https://www.ncbi.nlm.nih.gov/pmc/articles/PMC7005461/>
7. <https://pubmed.ncbi.nlm.nih.gov/35382010/>

Reviewer #2: Thankyou for giving me an opportunity to review this. The title of the study is quite attractive but the findings are very limited and not well explained. On the other hand, the methodology is an important part of any study and in this study, methodology is not cleared. There is no sampling techniques, no rules for sample size calculation and sample size is not justified. Also there is no clarity about participation rate. Most importantly, discussion part is not satisfied and not elaborate all the important points of this study. I appreciate your efforts. But improvement is needed. Thankyou.

In the Materials and methods section, the methodology has been clarified, the sample techniques have been justified, the participation rate has been explained.
