# Supplementary material for: Health-related behavioral changes during the COVID-19 pandemic. A comparison between cohorts of French and Italian university students
Source: PLOS Glob Public Health. 2023 Sep 8;3(9):e0002298. doi: 10.1371/journal.pgph.0002298 (PMC10490880; doi:10.1371/journal.pgph.0002298)
Supplement: Attachment — Submitted filename: Rebuttal Letter 20-7-23.docx [file pgph.0002298.s004.docx]

**Rebuttal Letter 20/07/2023**

Journal Requirements:

1. Please review your reference list to ensure that it is complete and correct. If you have cited papers that have been retracted, please include the rationale for doing so in the manuscript text, or remove these references and replace them with relevant current references. Any changes to the reference list should be mentioned in the rebuttal letter that accompanies your revised manuscript. If you need to cite a retracted article, indicate the article’s retracted status in the References list and also include a citation and full reference for the retraction notice.

I reviewed my reference list. It is complete and correct. No changes have been made involving the addition or removal or correction of cited papers.

Additional Editor Comments (if provided):

Thank you for your attention to the comments raised by prior reviewers. I believe most of them have been addressed adequately. However, there are remaining issues that should be addressed for the article to meet the journal’s publication criteria for rigor.

- Given that the study used a cross-sectional study design and the analysis involves descriptive statistics, the causal claims throughout the manuscript are not supported by adequate evidence. Please go through the entire manuscript and delete or modify the text so that no causal inference is made. For example, on Page 3, you state: Page 3 “Italian students suffered more than French students in their well-being due to the harder social restrictions applied in Italy”. However, your analysis does not specifically isolate “social restrictions” as a causal factor independent of confounders that might explain the different results between the two countries. These types of causal conclusions are found in several places in the manuscript.

The text of the entire manuscript has been deleted or modified to avoid causal inferences:

On page 3 I changed the text:

**From** “Italian students suffered more than French students in their well-being due to the harder social restrictions applied in Italy”

**To** “the health emergency may have played an active role in negatively impacting certain behaviors in both cohorts of university students”

In the Results, section Diet, I cancelled:

“Again, in this case as well, restrictions on going out may have had a direct effect”.

In the Discussion I deleted causal conclusions and revised the text:

“Nevertheless, the present analysis doesn’t isolate ‘social restriction’ as an independent causal factor, that permit causal inference; in addiction, hypothesis regarding other possible causes are included”.

I intervened in other point along the text to reduce or delete the cited causal inference.

- In the Methods, you state that ethical review is not required for human participants. However, research ethics boards are specifically designed to provide oversight on human subjects research. Please explain.

In the Method (ethics) I added:

“In fact the data collection is completely anonymous and data are not described except in an aggregated way, this makes the data sources (students) unrecognizable. Furthermore, the questionnarire explicitly stated that the compilation automatically implied the release on the use of the data provided in the above modalities”.

- In the Abstract, you use the term “prevalently” to describe the study design. I believe this is not a standard term. Perhaps you mean “cross-sectional”? Please change to clarify the meaning of this term/sentence.

In the Abstract I changed the term “prevalentely” in “cross-sectional”.

- The Methods section has been greatly improved in this version. However, it is still unclear how the students were selected for recruitment. Were all students in the directory contacted via email? How many students were attempted? What proportion were contacted? How many refused after contact?

In the Method section (Methodology) I added:

“The students were contacted via the institutional mailing list with a message without a request of confirmation of reading (again for privacy), so the number of emails that were sent is known, but not the number of students who actually read them. For this reason, the indicated response rates represent a lower bound on the participation of student cohorts. That is, the real rate was equal to or higher than the one indicated.”

- Please make sure your manuscript follows the STROBE guideline for cross-sectional observation studies: <https://www.strobe-statement.org/checklists/>

Having read the indication of the STROBE guidelines, I declare that, where possible, the indicated criteria have been followed. In particular:

10. In the survey design, the sample size was not fixed, because we drew on the entire courts available and compatible with a comparison between the two contexts.

12. The number of non-responses is very small and for this reason no specific analysis has been dedicated to them.

13.c We were unable to check the non-respondents as the data confidentiality method did not allow us to know the identity of the non-respondents, and therefore their characteristics.

- Please discuss the following points in the limitation section
o It’s possible that health behavior was affected not just by public health restrictions, but also having had family members or friends who died or were severely sick due to COVID. And also exposure to severe illnesses and rising mortality in the news. It seems that your analysis does not distinguish these and other factors that could affect health behavior outcomes. Please discuss this limitation.
o The low participation rates are major limitations. Please include a discussion of this.

In the Limitation section I added:

“It’s possible that health behavior was affected not just by public health restrictions, but also having had family members or friends who died or were severely sick due to COVID, as well as exposure to severe illnesses and rising mortality in the news”.

“The low participation rates are also major limitations”.
